# Alterations in mitochondrial DNA content in canine heart failure

**DOI:** 10.1186/s12917-025-04856-z

**Published:** 2025-07-01

**Authors:** Chavalit Boonyapakorn, Julalak Meechai, Panida Sansuktaweesup, Pitchaporn Karaket, Worawan Woravuthvitaya, Suphakan Chirathanaphirom, Phongsakorn Chuammitri, Chanon Piamsiri, Wanpitak Pongkan

**Affiliations:** 1 Faculty of Veterinary Medicine, Chiang Mai University, Chiang Mai, 50100 Thailand; 2 Small Animal Hospital, Chiang Mai University, Chiang Mai, 50100 Thailand; 3 Integrative Research Center for Veterinary Circulatory Sciences, Faculty of Veterinary Medicine, Chiang Mai University, Chiang Mai, 50100 Thailand; 4 Research Center for Veterinary Biosciences and Veterinary Public Health, Faculty of Veterinary Medicine, Chiang Mai University, Chiang Mai, 50100 Thailand

**Keywords:** Heart failure, Dogs, Mitochondrial DNA content

## Abstract

**Background:**

Heart failure is the most common lethal consequence of cardiovascular abnormalities in the dog population. In humans, the abundance of mtDNA content plays a crucial role in the pathogenesis of different types of cardiovascular diseases such as ischemic heart disease, dilated cardiomyopathy, and congestive HF. Changes in mtDNA copy number could indicate the extent of mtDNA damage, serving as a potential biomarker for mitochondrial function and a predictor of several cardiovascular disease risks in humans. However, evidence regarding the alterations in mtDNA content in canine heart failure remains poorly explored. This study aims to determine the peripheral blood mitochondrial DNA content in healthy dogs and those experiencing acute heart failure.

**Results:**

A total of thirty client-owned dogs, aged between 5 and 15 years, were selected for the study. The dogs were categorized into two groups: a healthy group (HT, *n* = 15) and a heart failure group (HF, *n* = 15). A comprehensive evaluation was performed on all dogs, involving physical examination, thoracic radiography, and transthoracic echocardiographic assessment. Additionally, three milliliters of blood were collected for hematology, blood chemistry profiles, and mitochondrial DNA (mtDNA) analysis. The monocyte levels were found to be significantly higher in the heart failure group (HF) than in the healthy group (HT). Furthermore, parameters such as blood urea nitrogen (BUN), creatinine, vertebral heart score (VHS), and vertebral left atrial size (VLAS) showed significant elevation in the HF group (p-value < 0.05). Echocardiographic measurements, including left ventricular internal dimension at end-diastole (LVIDd), left atrium (LA), left atrial to aortic root ratio (LA/Ao), end-diastolic volume (EDV), end-systolic volume (ESV), and left ventricular mass, were also notably higher in the HF group compared to the healthy group (p-value < 0.05). Moreover, the mtDNA content was significantly higher in the HF group than in the healthy group (p-value < 0.05).

**Conclusion:**

In the context of decompensated HF, the occurrence of tissue hypoxia might instigate cellular damage, consequently resulting in the release of mtDNA. This phenomenon potentially explains the observed higher mtDNA content in the HF group in comparison to the healthy group.

## Background

In the field of small animal medicine, various cardiac conditions culminate in a complex syndrome known as heart failure (HF), resulting in significant financial burdens and high mortality rates. HF is characterized by the heart’s inability to adequately circulate blood to sustain the body’s tissues. Given the critical role of mitochondria in powering cardiac function, there is a growing interest in investigating potential mitochondrial dysfunction within the context of HF. Notably, a study conducted by Clay Montier et al. (2009) revealed an upsurge in energy demand and oxidative stress within cardiomyocytes during HF, leading to cellular damage triggered by heightened levels of reactive oxygen species (ROS) and subsequent reduction in mitochondrial DNA (mtDNA), a process closely associated with cellular aging [1]. Furthermore, increased oxidative stress has been found to induce mutations in mtDNA [2, 3].

The heart, being a high-energy-demanding organ, heavily relies on mitochondria as its primary energy source. According to Anastacio et al. (2013), approximately 29–36% of the volume of myocytes is occupied by mitochondria, rendering it the cell type with the highest mitochondrial density [4]. These mitochondria actively facilitate the production of adenosine triphosphate (ATP) through the oxidative phosphorylation process, providing crucial support for the heart’s essential functions of contraction and relaxation [5].

Moreover, beyond their role in energy production, mitochondria also serve as significant sites for the generation of reactive oxygen species, regulators of cellular calcium ion balance, and mediators of myocardial apoptosis [5, 6]. Since adult mammalian cardiomyocytes lack the ability to replicate and replace themselves [7, 8], the sufficient levels of injuries or pathologies would affect overall function in both organs, cellular, and interorganelle levels. Previous studies have reported the impairment of peripheral blood mononuclear cell (PBMC) mitochondrial bioenergetic and mitochondrial respiration in dogs with myxomatous mitral valve disease (MMVD) [9, 10]. In human, the impairment of mitochondrial functions has been reported to associated with cardiac contractile dysfunction and subsequent HF [11, 12].

Previously, it has been demonstrated a positive correlation between peripheral blood mitochondrial mtDNA content and myocardial mtDNA levels in patients with end-stage HF who underwent cardiac transplantation [13]. Additionally, several reports have highlighted significantly elevated levels of peripheral blood mtDNA in HF patients compared to healthy individuals [14]. However, within the domain of veterinary medicine, there remains a lack of investigation on mtDNA content in dogs with HF. This study aims to uncover potential differences in peripheral blood mtDNA levels between dogs with HF and their healthy counterparts. It is anticipated that the findings from this study could offer valuable insights for the development of improved diagnostic methods and treatment strategies for HF within the veterinary field in the future.

## Methods

### Study populations

Client-owned dogs, both males and females, within the age range of 5 to 15 years, were recruited from the cardiology clinic at the Small Animal Veterinary Teaching Hospital, Faculty of Veterinary Medicine, Chiang Mai University. The recruited dogs were categorized into two distinct groups: the healthy group and the HF group. In this study, dogs in HF groups consisted of ACVIM stages C and D patients who received standard treatments while they were experiencing their episode of acute decompensated HF. In contrast, the non-HF group included clinically healthy dogs of breeds predisposed to HF. Based on previous medical records, current physical examination, and blood work, dogs with a recent or chronic history of decreased appetite, vomiting, or diarrhea or that had a suspicion of active pancreatitis, and chronic inflammatory diseases such as arthritis, dermatitis, inflammatory bowel disease, hepatopathy, cholangitis, and respiratory issues were be excluded from the study. Dogs with known concurrent conditions (e.g. neoplasia) or other reasons that decreased the likelihood of completing the study were also excluded. Blood samples for mitochondrial DNA extraction were taken after history taking and routine physical examination. The assessment for HF was entail a thoracic radiography, which is expected to reveal manifestations such as pulmonary venous distension and interstitial or alveolar infiltration in the perihilar area extending to the caudodorsal lung lobe.

### Physical examination

All enrolled dogs underwent a comprehensive physical examination, during which baseline data were systematically collected. This data encompasses various parameters, including body weight, body temperature, body condition score, mentation, heart rate, heart sounds, pulse rate, pulse quality, respiratory rate, lung sounds, hydration status, mucous membrane colour, capillary refill time, and palpation from head to tail. Furthermore, a thorough heart auscultation was conducted, encompassing examination of all four valve areas [15]. In particular, any murmurs in the heart sounds were documented using a six-scale system [16]. This meticulous examination enable a detailed assessment of the dogs’ overall health and cardiac condition.

### Thoracic radiography

In this study, right lateral and dorsoventral thoracic radiographs were obtained from all participating dogs to assess the cardiac silhouette, airway, and lung parenchyma. The vertebral heart scale (VHS) and vertebral left atrial size (VLAS) were quantified based on the right lateral radiograph. VHS was calculated by establishing the cardiac long axis, which extends from the ventral aspect of the carina to the cardiac apex. The short axis was measured by drawing a line perpendicular to the long axis. The measurements of the long axis and short axis were then projected to the starting point of the fourth thoracic vertebrae. The count of vertebrae spanned by this measurement defined the VHS [17]. Additionally, the VLAS was determined by drawing a line from the ventral aspect of the carina to the caudal aspect of the left atrium, where it intersects with the dorsal border of the caudal vena cava. This line was then transposed to the cranial edge of the fourth thoracic vertebrae, and the number of vertebrae spanned by this measurement quantified the VLAS [18, 19].

### Echocardiography

Transthoracic echocardiography was conducted with the dogs positioned in right lateral recumbency, without the use of sedation. This approach allowed for the thorough evaluation of cardiac structures, including valvular structures, using a two-dimensional (2D) view. The assessment of left ventricular dimensions and function was achieved through the utilization of M-mode echocardiography. Additionally, the left atrial size to aortic root ratio was determined using the Swedish method [20]. The transthoracic echocardiography was assessed as a blind investigation by two experienced cardiologists to ensure an unbiased evaluation.

### Blood sample collection

A total of three milliliters (3 mL) of blood was collected from either the cephalic or saphenous vein of the dogs by venipuncture procedure under aseptic technique consideration. The collected blood samples were divided into two portions. The initial one milliliter (1 mL) of blood was carefully transferred into an EDTA tube, which served two purposes: hematology analysis utilizing a Hematology Analyzer (BC-5300Vet, Mindray^®^, China) and mitochondrial DNA extraction from the whole blood. The EDTA blood was stored at -20 ^O^C for further mitochondrial DNA extraction.

The subsequent two milliliters (2 mL) of blood were deposited into a heparin tube, designated for routine serum biochemistry assessments. These assessments encompassed the analysis of parameters such as creatinine, blood urea nitrogen (BUN), alanine transaminase (ALT), alkaline phosphatase (ALP), total protein, and albumin. These biochemical evaluations were carried out using an Automated Chemistry Analyzer (BX-3010, Symes^®^).

### Mitochondrial DNA extraction and mitochondrial DNA content analysis

Before mitochondrial DNA (mtDNA) extraction, EDTA blood samples were removed from storage at − 20 °C and thawed at room temperature [21]. mtDNA extraction from the whole blood collected in the EDTA tube was performed using the NucleoSpin^®^ Tissue kit (Macherey-Nagel, Dueren, Germany), as per the manufacturer’s recommendation and protocols described in previous studies [21, 22]. In brief, samples were prepared in 180 µL T1 buffer and 25 µL proteinase K for 3 h at 56 °C. Samples were lysed in 200 µL of B3 buffer for 10 min at 70 °C. After adding 210 µL absolute ethanol, the samples were transferred to the NucleoSpin Tissue columns and centrifuged at 11,000 g for 1 min. The silica membranes were washed twice with 500 µl of Buffer BW, each followed by centrifugation at 11,000 g for 1 min. The columns were centrifuged again to remove any remaining ethanol. Finally, DNA was eluted using 30 µl of TE buffer [21, 22].

For mitochondrial DNA content analysis, the glyceraldehyde-3-phosphate dehydrogenase (GAPDH) gene was employed as a housekeeping gene [21]. The sequence of the forward and reverse primers for GAPDH, with a melting temperature of 59 °C, was designed as indicated in Table 1. Additionally, the mitochondrial-encoded NADH-ubiquinone oxidoreductase subunit 4 (Mt-ND4) was amplified to assess mitochondrial DNA in this study. The sequences of the forward and reverse primers for Mt-ND4 are outlined in Table 1 [23].

**Table 1:** The sequence of forward and reverse primers of GA-3-PDH and Mt-ND4

mtDNA	sequence
GAPDH	5’-TCACCAGGGCTGCTTTTAAC-3’
	5’-TGACTGTGCCGTGGAATTTG-3’
Mt-ND4	5’-CGTAATCAGTCCCGTAGGTGTTAGA-3’
	5’-ACATTAGCCAGCATGATACCAATCG-3’

Abbreviation: GAPDH, Glyceraldehyde-3-phosphate dehydrogenase; Mt-ND4, mitochondrially encoded NADH: ubiquinone oxidoreductase core subunit 4; mtDNA, mitochondrial DNA

Quantitative real-time PCR (qPCR) was employed to analyze the mitochondrial DNA (mtDNA) content in the samples. For this analysis, the HOT FIREPol^®^ EvaGreen^®^ qPCR Mix Plus (ROX) from Solis BioDyne, Estonia, was utilized as the master mix. The PCR protocol involved an initial denaturation step at 95 °C for 12 min, followed by annealing at 95 °C for 15 s and extension at 60 °C for 15 s.

In each sample, the cycle threshold (Ct) value of both the GAPDH and Mt-ND4 genes was determined. To assess the relative mtDNA content, normalization was carried out using the GAPDH gene as an endogenous control, employing the comparative Ct method [24]. This normalization method enables the comparison of the expression levels of the target Mt-ND4 gene with the reference GAPDH gene, facilitating a precise evaluation of the mtDNA content in the context of HF in the canine subjects.

### Statistical analysis

In our study, the Shapiro-Wilk test was used to assess the normality of the dataset. All variables were found to be normally distributed; therefore, statistical comparisons between groups were performed using independent t-tests. The presentation of parametric data were presented as mean ± standard error (mean ± SE). In contrast, non-parametric data such as sex, lung sounds, and heart sounds were summarized descriptively as percentages using descriptive statistics. All statistical analyses and calculations were performed using the R Studio Software, version 1.2.5033. For all statistical tests and analyses, significance was considered at a threshold of p-value less than 0.05.

## Results

### Demographic characteristics of sample groups

The HF group comprised 14 dogs with MMVD (93.33%) and 1 dog with DCM (6.64%). The average age of the HF group was significantly higher than that of the healthy group (p-value < 0.01). The HF group consisted of 6 female dogs (40.00%) and 9 male dogs (60.00%), while the healthy group consisted of 10 female dogs (66.67%) and 5 male dogs (33.33%). Regarding breeds, the healthy group included 5 Pomeranians, 4 Chihuahuas, 1 French Bulldog, and 1 Pekingese, while the HF group included 7 Mixed breeds, 2 Chihuahuas, 2 Shih Tzus, 1 French Bulldog, 1 Golden Retriever, 1 Poodle Toy, and 1 Bang Kaew. When comparing the mean body weight between the sample groups, it was found that the body weight of the HF group was significantly higher than that of the healthy group (p-value < 0.01, Table 2).

**Table 2:** Demographic characteristics of dogs in healthy and heart failure groups included age, sex, breed, and body weight

	Healthy group (*n* = 15)	Heart failure group (*n* = 15)
Age (years)	9.02 ± 3.07	11.42 ± 2.10*
Sex, n, male (%)	5 (33.33%)	9 (60%)
Breed, n • Chihuahua • Shih tzu • French bulldog • Golden retriever • Mixed breed • Poodle toy • Bangkaew • Pomeranian • Beijing	4 3 1 0 0 1 0 5 1	2 2 1 1 7 1 1 0 0
Body weight (kilograms)	4.83 ± 2.40	12.32 ± 9.12*

Age and body weight were presented as mean ± SD. While Sex and breed were presented as numbers with percentages. * Statistical significant difference between healthy and heart failure groups

### Physical examination findings in the healthy and heart failure groups

In this study, we found that HF dogs had a significant increase in heart rate and respiratory rate compared to the healthy group (Table 3). Crackle lung sound has been detected in some of the HF dogs (i.e. 3/15 dogs) while capillary refill time was still normal (i.e. less than 2 s) in both groups. All dogs in HF groups were recorded with abnormal heart sounds (i.e. muffle heart sound 1/15 dogs and murmur heart sound 15/15 dogs) during physical examination.

**Table 3:** Physical examination of dogs in the healthy and heart failure groups

	Healthy group (*n* = 15)	Heart failure group (*n* = 15)	*p*-value
Heart rate (BPM)	119.75 ± 25.76	141.111 ± 31.00*	0.0045*
Respiratory rate (RPM)	21.1 ± 14.97	44.000 ± 11.55*	< 0.001*
Capillary refill time (Sec)	< 2	< 2	NA
Lung sound, n (%)	Normal 15/15 (100%)	Normal 12/15 (80%)	NA
Heart sound, n (%)	Normal 15/15 (100%)	Abnormal 15/15 (100%)	NA

* Statistical significant difference between healthy and heart failure groups

### Hematology and blood chemistry profile in the healthy and heart failure groups

A complete blood count revealed a significantly higher monocyte count in the HF group compared to the healthy group (p-value = 0.04, Table 4). Other hematology profiles did not show a statistical difference between the healthy and HF groups. Blood chemistry profiles indicated that the blood urea nitrogen (BUN) and creatinine levels in the HF group were significantly higher than those in the healthy group (p-value < 0.01 and < 0.01, respectively), while the albumin level in the HF group was significantly lower than that in the healthy group (p-value = 0.02, Table 4).

**Table 4:** Hematology and blood biochemistry profile in the healthy and heart failure groups

	Healthy group (*n* = 15)	Heart failure group (*n* = 15)	*p*-value
Hematology profile			
• Hematocrit (%)	50.01 ± 5.58	46.14 ± 6.60	0.1577
• RBC (x10^6^/µL)	7.22 ± 0.83	6.88 ± 0.88	0.5730
• WBC (cell/µL)	10,902.00 ± 3570.85	13,626.00 ± 5250.62	0.0730
• MCV (fL)	68.934 ± 2.43	67.089 ± 5.74	0.0673
• MCH (pg)	24.040 ± 0.67	22.956 ± 2.50	0.8432
• MCHC (g/dL)	34.550 ± 0.57	34.167 ± 1.21	0.3542
• Hgb (g/dL)	15.311 ± 1.92	16.93 ± 1.24	0.4025
• Neutrophil (cell/µL)	7,674.00 ± 3,189.68	10,488.00 ± 4,321.25	0.2565
• Lymphocyte (cell/µL)	1,839.00 ± 738.07	1,390.00 ± 534.61	0.2259
• Monocyte (cell/µL)	690 ± 337.41	873.00 ± 703.71*	0.0027*
• Eosinophil (cell/µL)	683.30 ± 514.65	736.40 ± 455.34	0.1581
• Basophil (cell/µL)	26.00 ± 55.62	32.86 ± 34.50	0.1475
• Platelet (10^3^cell/µL)	398.125 ± 78.17	466.286 ± 75.25	0.3168
Biochemistry profile			
• BUN (mg/dl)	18.79 ± 6.08	65.34 ± 56.42*	< 0.0001*
• Creatinine (mg/dl)	0.88 ± 0.20	1.76 ± 0.79*	0.0130*
• ALT (U/L)	80.93 ± 51.86	93.80 ± 122.02	0.2549
• ALP (U/L)	105.5 ± 155.69	284.90 ± 327.15	0.1981
• Total protein (g/dL)	7.74 ± 0.78	7.52 ± 0.77	0.1768*
• Albumin (g/dL)	3.52 ± 0.33	3.12 ± 0.53*	0.0100*

The value was presented as mean ± SD. * Statistical significant difference between healthy and heart failure groups

### Thoracic radiography in the healthy and heart failure groups

Thoracic radiography revealed a highly significant increase in both the vertebral heart scale (VHS) and vertebral left atrial size (VLAS) in the HF group when compared to the healthy group (p-value < 0.01 and < 0.01, respectively, Table 5).

**Table 5:** The radiographic findings, including vertebral heart scale (VHS) and vertebral left atrial size in healthy and heart failure groups

	Healthy group (*n* = 15)	Heart failure group (*n* = 15)	*p*-value
VHS	9.70 ± 0.69	12.13 ± 1.45*	< 0.01*
VLAS	2.57 ± 0.37	3.41 ± 0.65*	< 0.01*

The value was presented as mean ± SD.* Statistical significant difference between healthy and heart failure groups

### Echocardiographic analysis

In the echocardiographic results, the average values of cardiac dimension parameters, including left ventricular internal diameter in diastole (LVIDD, *p* < 0.001), left ventricular posterior wall in diastole (LVPWD, *p* = 0.049), interventricular septal in systole (IVSS, *p* = 0.005), left ventricular internal diameter in systole (LVIDS, *p* = 0.001), left ventricular posterior wall in systole (LVPWS, *p* = 0.004), end-diastolic volume (EDV, *p* < 0.001), end-systolic volume (ESV, *p* = 0.001), aortic root diameter (AoR, *p* = 0.038), left atrial diameter (LA diameter, *p* < 0.001), and left atrial to aortic root ratio (LA/Ao, *p* < 0.001) in HF dogs were significantly higher than those in healthy dogs. The normalized left ventricular internal dimension during diastole (LVIDDN) and systole (LVIDSN) was also augmented among those HF dogs. (Table 6) However, cardiac function parameters, including the percentage of ejection fraction (EF) and the percentage of fractional shortening (FS), did not show a statistical difference between healthy and HF dogs. Collectively, these echocardiographic findings suggest that HF dogs exhibited an eccentric cardiac remodelling phenotype while maintaining preserved systolic function.

**Table 6:** Comparing echocardiography analysis results between the healthy and heart failure groups

	Healthy group (*n* = 15)	Heart failure group (*n* = 15)	*p*-value
IVSD (cm)	0.67 ± 0.14	0.75 ± 0.13	0.4025
LVIDD (cm)	2.08 ± 0.45	3.98 ± 1.45*	< 0.0001*
LVPWD (cm)	0.60 ± 0.16	0.72 ± 0.22*	0.0039*
IVSS (cm)	0.92 ± 0.18	1.21 ± 0.41*	0.0007*
LVIDS (cm)	1.24 ± 0.42	2.28 ± 1.27*	0.0232*
LVPWS (cm)	0.92 ± 0.25	1.21 ± 0.30*	0.0377*
LVIDDN (mm)	1.304 ± 0.25	2.163 ± 0.13*	0.0107*
LVIDSN (mm)	0.805 ± 0.13	1.100 ± 0.34*	0.0012*
ESV (ml)	4.55 ± 3.70	27.00 ± 32.03*	0.0073*
EF (%)	74.09 ± 11.09	75.69 ± 14.78	0.1384
FS (%)	41.81 ± 10.04	46.10 ± 13.59	0.4805
AoR (cm)	1.12 ± 0.23	1.38 ± 0.39*	0.0021*
LA diameter (cm)	1.49 ± 0.36	3.53 ± 1.27*	0.0032*
LA/Ao	1.35 ± 0.20	2.59 ± 0.73*	< 0.0001*

* Statistical significant difference between healthy and heart failure groups. IVSD; Interventricular septal end diastole, LVIDD; Left ventricular internal dimension in end diastole, LVPWD; Left ventricular posterior wall end diastole, IVSS; Interventricular septal end systole, LVIDS; Left ventricular internal dimension in end systole, LVPWS; Left ventricular posterior wall end systole, LVIDDN; normalized left ventricular internal dimension during end diastole, LVIDSN; normalized left ventricular internal dimension during end systole, EDV; End diastolic volume, ESV; End systolic volume, EF; Ejection fraction, FS; Fractional shortening, AoR; Aortic root diameter, LA diameter; Left atrium diameter, LA/Ao; Left atrial to aortic root ratio.

### Mitochondrial DNA (mtDNA) content analysis

**Fig. 1:**
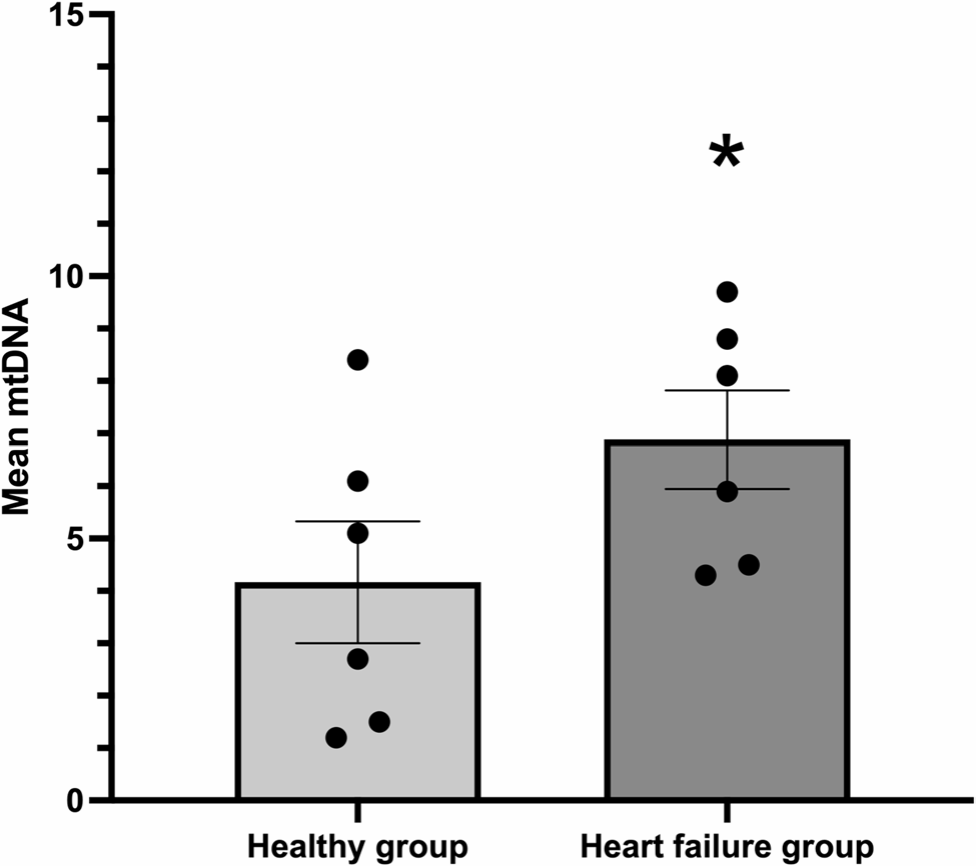
The mtDNA content in healthy and heart failure groups. * Statistical significant difference between healthy and heart failure groups

The mtDNA content in the HF group was significantly higher than in the healthy group (p-value = 0.03). Specifically, the mean mtDNA content was 3.96 ± 3.81 in the healthy group and 6.48 ± 3.45 in the HF group (Table 7; Fig. 1).

**Table 7:** The MtDNA content between the healthy and heart failure groups

	Healthy group (*n* = 15)	Heart failure group (*n* = 15)	*p*-value
mtDNA content	3.96 ± 3.81	6.48 ± 3.45*	0.0143*

* Statistical significant difference between healthy and heart failure groups. Data were presented as mean ± standard error (mean ± SE)

## Discussion

HF is the most common lethal consequence of cardiovascular abnormalities in dog population [21, 25]. Previously, the overwhelming intracellular and mitochondrial oxidative stress during HF has been reported to cause cardiac mitochondrial damage and dysfunction, which are subsequently degraded through the mitophagy process [3, 26]. However, mtDNA that escapes mitophagy remains physiologically active as a damage-associated molecular pattern (DAMP), potentially being released into the extracellular compartment and triggering further undesirable events [27, 28]. In human, previous studies reported that the abundance of mtDNA content plays a crucial role in the pathogenesis of different types of cardiovascular diseases such as ischemic heart disease, dilated cardiomyopathy, and congestive HF [27, 29]. Additionally, it has been reported the potential utilization of mtDNA copy numbers to predict the occurrence, development, and prognosis of cardiovascular outcomes [27]. Changes in mtDNA copy number could indicate the extent of mtDNA damage, serving as a potential biomarker for mitochondrial function and a predictor of several cardiovascular disease risks in humans [27, 29]. However, evidence regarding the alterations in mtDNA content in canine HF remains limited and poorly explored. In this study, we compared mtDNA content, demographic characteristics, blood work, and cardiac function in dogs with HF secondary to MMVD and DCM, relative to healthy control animals. The major finding in this study was included with (i) there was a significant worsening of clinical characteristics, including hematology, biochemistry, radiography, and echocardiographic profiles, in dogs with HF secondary to both MMVD and DCM, compared to healthy dogs. (ii) There was a significant escalation in mtDNA content in dogs with HF compared to healthy dogs.

As previously reported, MMVD is considered the most dominant cardiovascular abnormality in the dog population [30, 31]. MMVD is a chronic degeneration of the atrioventricular valves. The progression of valve lesions and increased valve regurgitation can lead to a loss of forward stroke volume, reduced contractile force, adverse remodeling of the left atrium and left ventricle, cardiac hypertrophy and dilation, elevated heart rate, and subsequent left-sided HF [30, 31]. In line with these notions, we also found that the clinical characteristics between healthy and HF dogs were different, especially in body weight, which was significantly higher in HF dogs. Whereas the HF dogs consisted of medium to large breeds, healthy dogs were mostly small breeds. Our echocardiogram results showed a significant increase in LVPWD and LVPWS resulting from different breeds and body weight, which affected the cardiac dimensions measured from the M-mode echocardiogram [32, 33]. Besides, the left ventricular end-diastolic volume and end-systolic volume (EDV and ESV) were significantly higher in HF dogs, possibly affected by remodeling of cardiac structure and left ventricular volume overload secondary to the underlying HF. In addition to the conventional echocardiographic assessment, utilizing the advanced imaging techniques (i.e., myocardial work, Doppler echocardiography, and speckle-tracking echocardiography) in future investigations could provide a more comprehensive evaluation of myocardial performance and could significantly strengthen the clinical relevance and application value.

The majority of circulating cell-free DNA has two major subpopulations, including mitochondrial-derived cell-free DNA (cf-mtDNA) and nuclear-derived cell-free DNA (cf-nDNA) [34, 35]. mtDNA contents originate from two primary sources, including the liberated mtDNA pool, which is released from the damaged cells (e.g., cardiomyocytes, leukocytes, lungs, and myocytes) in response to tissue injury or stress, and peripheral white blood cell mtDNA [21, 36]. Meanwhile, cell-free nuclear DNA (nDNA) was also reported to be released into systemic circulation in response to non-selective cellular membrane permeability, cellular damage, or cell death in several diseases, including heart failure [34, 35, 37]. In humans, both mtDNA and nDNA have been shown to have predictive value for diagnosis and treatment outcomes in cardiovascular diseases [34, 37, 38]. Dhondup and colleagues (2016) proposed that elevated plasma levels of mtDNA and nDNA may result from increased net release by the failing myocardium, with additional contributions from damage to other organs, potentially further increasing circulating cell-free DNA levels [39]. Concerning these notions, further studies are warranted by the inclusion of nDNA as a reference to estimate mtDNA content could improve the precision and reliability of the measurements. Future studies incorporating cf-nDNA quantification alongside cf-mtDNA could offer a more comprehensive understanding of the pathophysiological processes in heart failure and enhance the biomarker potential of cell-free DNA profiles. More interestingly, our data showed that the mtDNA content was significantly higher in dogs with HF than in healthy dogs. This finding aligns with a study in chronic HF patients compared to healthy individuals, where HF patients demonstrated an increase in peripheral mtDNA content, which correlated with the severity of the disease [39]. In addition, a previous study in humans demonstrated that mtDNA content in acute HF patients was higher than the chronic HF patients [14]. Consistently, a recent study from our group has demonstrated that mtDNA content was significantly higher in MMVD stage C and D compared to the stage B group [21]. Additionally, previous studies in both human and preclinical rat models have shown that mtDNA levels are elevated during stable and decompensated phases of heart failure with preserved ejection fraction due to chronic negative energy balance, exaggerated by cardiac dysfunction [36, 40]. The observed increase in mtDNA content during HF might result from hypoperfusion, leading to cellular hypoxia and cell death, subsequently releasing mtDNA content into the bloodstream [14, 39]. These findings suggest that mtDNA levels may serve as a promising novel biomarker for canine heart failure, particularly in cases of MMVD, within clinical settings. With further validation, mtDNA could contribute to earlier diagnosis, improved disease monitoring, and more targeted therapeutic strategies, ultimately enhancing clinical outcomes in affected dogs. The previous findings from our group demonstrated that mean malondialdehyde (MDA) levels, which are a marker for oxidative byproducts, in the MMVD stage C group tended to be higher compared to healthy controls, although the difference was not statistically significant [21]. This is consistent with another study, which also found no statistically significant differences in oxidative stress markers (i.e., plasma MDA and oxidized low-density lipoprotein [oxLDL]) or antioxidative markers (i.e., α-tocopherol and γ-tocopherol) between MMVD with HF and non-HF dogs [41]. Based on these findings, we propose that further studies measuring mitochondrial ROS (mtROS) levels using isolated mitochondria from myocardial tissue or peripheral blood mononuclear cells (PBMCs) should be warranted to provide more specific insights into myocardial oxidative status in MMVD dogs.

However, our results were inconsistent with the previous study that measured mtDNA content from PBMC in humans, which showed a significantly higher mtDNA content in PBMC from HF patients compared to healthy individuals [13]. We propose that the inconsistent findings could be due to the distinct sources of mtDNA. Since our study extracted mtDNA from whole blood samples, it is possible that the mtDNA originated from two sources: peripheral blood cells and the circulating mtDNA in plasma, released from dead cells [14, 39]. The decrease in mtDNA extracted from peripheral blood cells resulted from the oxidative stress occurring during HF [42]. It has been shown that oxidative stress can damage mitochondria, leading to subsequent released of mtDNA into the extracellular space [43, 44]. Although our study did not include data comparing mtDNA content in acute and chronic HF dogs, Krychtiuk et al. (2020) showed that mtDNA content was significantly higher in acute HF patients compared to those with chronic HF [14]. In contrast, Anguita et al. (2022) has reported the significant higher in mtDNA content from PBMC in chronic HF patients compared to those acute HF patients. They proposed that this increment might be a compensatory process during chronic HF progression [45]. A well-designed prospective study investigating mtDNA levels in dogs with MMVD-associated heart failure, particularly before and after recommended medical treatments, could provide important insights into disease mechanisms, treatment responses, and prognostic value. Such findings could ultimately support the development of mtDNA as a reliable biomarker for early detection, therapeutic monitoring, and outcome prediction in clinical practice.

Moreover, it has been shown that mtDNA can directly bind to toll-like receptor (TLR) 6 and 9, which subsequently leads to the activation of NOD-, LRR- and pyrin domain-containing protein 3 (NLRP3) inflammasome, resulting in the upregulation and release of pro-inflammatory cytokines [46–49]. In this study, we found the evidence of systemic inflammation reflected by the significant increase in monocyte count among the HF dogs compared to those healthy animals. However, we did not investigate the pro-inflammatory biomarkers (e.g., interleukin-1 beta or IL-1β, interleukin 6 or IL-6, tumor necrosis factor alpha or TNF-α) in this study. In line with our notion, previous studies have also shown an increase in monocyte count along with elevated inflammatory biomarkers in HF dogs [50, 51]. Likewise, a study in humans found that monocyte levels and inflammatory biomarkers increased during acute HF, then decreased to similar levels as healthy controls after treatment [52]. However, due to the limited volume of blood samples obtained from the animals, we were unable to assess cardiac injury biomarkers in this experiment. Nevertheless, a previous study from our group demonstrated that plasma levels of the cardiac injury biomarker N-terminal pro-B-type natriuretic peptide (NT-proBNP) were significantly elevated in dogs with MMVD-related HF [53]. Similarly, we observed that plasma levels of humanin, a mitochondria-derived peptide, were significantly decreased in heart failure groups compared to healthy controls [53]. Previous studies in humans have also shown that reduced systemic humanin levels are associated with mitochondrial injury, mitochondrial dysfunction, and adverse outcomes in patients with HF [54–56]. We propose that future studies investigating the relationship between cardiac injury biomarkers (e.g., the cardiac troponin and natriuretic peptide families) and mtDNA are warranted to confirm the specificity of mtDNA as a marker for cardiac injury and HF.

From the biochemistry profile, we observed a lower level of albumin in HF dogs than in healthy dogs. Numerous studies in humans have indicated that a lower level of albumin is associated with mortality and long-term outcomes in both acute and chronic HF patients [57–62]. Moreover, previous studies in humans found an association between low albumin levels and the inflammatory biomarker C-reactive protein (CRP) [59, 61], as well as higher blood urea nitrogen and creatinine levels [58, 59]. Precisely, Gotsman et al. (2019) suggested that the cause of low albumin levels during HF includes volume overload leading to hemodilution, hepatic congestion, chronic inflammation, malnutrition, and cachexia, all contributing to reduced albumin production [61]. Furthermore, inflammation can also induce albumin degradation [60]. The higher blood urea nitrogen and creatinine levels in HF dogs compared to healthy dogs can occur due to renal hypoperfusion or renal venous congestion during HF [63]. Additionally, a higher creatinine level can indicate a worse outcome after discharge from the hospital [64]. Notably, our blood chemistry demonstrates that the HF group was found to have azotemia. Supporting this notion, previous studies demonstrated that azotemia occurred concurrently in dogs with MMVD ACVIM stage B1, suggesting that renal damage may occur even prior to the development of HF [65, 66].

Our study is subject to certain limitations. Firstly, the sample size was relatively small, and there was a lack of matching in terms of breed, sex, and age between healthy and HF dogs, potentially influencing the mtDNA content. Secondly, the mtDNA source was extracted from whole blood, comprising both peripheral blood cells and free mtDNA in plasma. This dual origin may yield divergent results due to different pathogenic mechanisms. To address this, future investigations are warranted and should consider on separately examining plasma mtDNA content and mtDNA from peripheral blood cells. Additionally, in this study, dogs in the HF groups received standard treatments recommended by the ACVIM guideline [18] while they were experiencing episodes of acute decompensated HF. This could have introduced potential confounding effects from cardiac medications. However, a recent study from our group demonstrated that mtDNA levels declined during the subclinical stage of HF (ACVIM stage B) but increased markedly in more advanced stages (ACVIM stages C and D), despite also receiving standard medical treatment [21]. To eliminate the potential confounding effects of cardiac medications, we propose that future studies should specifically investigate acutely diagnosed, first-episode HF patients who have never received any form of cardiac treatment prior to sample collection. Moreover, incorporating analyses of inflammatory, oxidative stress, and cardiac injury biomarkers would contribute to a more comprehensive understanding of the pathogenesis of HF.

## Conclusion

In summary, our findings revealed a remarkable elevation in mtDNA content in dogs experiencing acute HF. This significant increase in mtDNA levels suggests a potential involvement of mtDNA during the progression of HF. Furthermore, these results underscore the potential utility of mtDNA as a novel biomarker for the detection and monitoring of HF in dogs. Our study encourages further investigation into the dynamic changes of mtDNA to better understand its role in the pathophysiology of HF and to explore its application in clinical diagnostics and prognostics for canine cardiovascular conditions.

## Data Availability

The data are available from the corresponding author upon reasonable request.

## Abbreviations

ACVIM: American College of Veterinary Internal Medicine
AoR: Aortic root diameter
EDV: End diastolic volume
EF: Ejection fraction
ESV: End systolic volume
FS: Fractional shortening
HF: Heart failure
HT: Healthy
IVSD: Interventricular septal end diastole
IVSS: Interventricular septal end systole
LA diameter: Left atrium diameter
LA/Ao: Left atrial to aortic root ratio
LVIDD: Left ventricular internal dimension in end diastole
LVIDDN: Normalized left ventricular internal dimension during end diastole
LVIDS: Left ventricular internal dimension in end systole
LVIDSN: Normalized left ventricular internal dimension during end systole
LVPWD: Left ventricular posterior wall end diastole
LVPWS: Left ventricular posterior wall end systole
MMVD: Myxomatous mitral valve disease
mtDNA: mitochondrial DNA
PBMC: Peripheral blood mononuclear cell
VHS: Vertebral heart score
VLAS: Vertebral left atrial size
